# Anticancer effect of minor phytocannabinoids in preclinical models of multiple myeloma

**DOI:** 10.1002/biof.2078

**Published:** 2024-05-17

**Authors:** Cristina Aguzzi, Laura Zeppa, Maria Beatrice Morelli, Oliviero Marinelli, Martina Giangrossi, Consuelo Amantini, Giorgio Santoni, Hossain Sazzad, Massimo Nabissi

**Affiliations:** ^1^ School of Pharmacy University of Camerino Camerino MC Italy; ^2^ Integrative Therapy Discovery Lab University of Camerino Camerino MC Italy; ^3^ School of Bioscience and Veterinary Medicine University of Camerino Camerino MC Italy; ^4^ Entourage Biosciences Inc. Vancouver Canada

**Keywords:** bone lesion, cell invasion, multiple myeloma, phytocannabinoids

## Abstract

Multiple myeloma (MM) is a blood cancer caused by uncontrolled growth of clonal plasmacells. Bone disease is responsible for the severe complications of MM and is caused by myeloma cells infiltrating the bone marrow and inducing osteoclast activation. To date, no treatment for MM is truly curative since patients relapse and become refractory to all drug classes. Cannabinoids are already used as palliative in cancer patients. Furthermore, their proper anticancer effect was demonstrated in many cancer models in vitro, in vivo, and in clinical trials. Anyway, few information was reported on the effect of cannabinoids on MM and no data has been provided on minor phytocannabinoids such as cannabigerol (CBG), cannabichromene (CBC), cannabinol (CBN), and cannabidivarin (CBDV). Scientific literature also reported cannabinoids beneficial effect against bone disease. Here, we examined the cytotoxic activity of CBG, CBC, CBN, and CBDV in vitro in MM cell lines, their effect in modulating MM cells invasion toward bone cells and the bone resorption. Subsequently, according to the in vitro results, we selected CBN for in vivo study in a MM xenograft mice model. Results showed that the phytocannabinoids inhibited MM cell growth and induced necrotic cell death. Moreover, the phytocannabinoids reduced the invasion of MM cells toward osteoblast cells and bone resorption in vitro. Lastly, CBN reduced in vivo tumor mass. Together, our results suggest that CBG, CBC, CBN, and CBDV can be promising anticancer agents for MM.

## Graphical abstract

Phytocannabinoids inhibit MM cell growth *in vitro* and in in vivo mouse model. Phytocannabinoids reduce MM cell invasion and resorption in bone in vitro model.

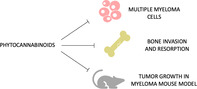

**Abbreviations**
CB1: cannabinoid receptor 1
CB2: cannabinoid receptor 2
CBC: cannabichromene
CBDV: cannabidivarin
CBG: cannabigerol
CBN: cannabinol
CTRL: control
DMSO: dimethyl sulfoxide
ECM: extracellular matrix
FBS: fetal bovine serum
GAPDH: glyceraldehydes‐3‐phosphate dehydrogenase
HEPES: N‐[2‐hydroxyethyl] piperazine‐N′‐[2‐ethanesulfonic acid]
HuOB: human osteoblast cell line
M‐CSF: macrophage colony stimulating factor
MM: multiple myeloma
MTT: 3‐[4,5‐dimethylthiazol‐ 2‐yl]‐2,5 diphenyl tetrazolium bromide
PI: propidium iodide
PAM: phenylacetylamide
PMA: phorbol‐12 myristate‐13 acetate
RANK‐L: receptor activator of nuclear factor κ‐B ligand
RPMI: RPMI8226
SD: standard deviation
SKO: SKO‐007
STR: short tandem repeat
THC: Δ^9^‐tetrahydrocannabinol
TRAP: tartrate‐resistant acid phosphatase
TRP: transient receptor potential
TRPV2: transient receptor potential vanilloid type‐2
VHC: vehicle

## 1 INTRODUCTION

A blood cancer, multiple myeloma (MM) occurs when clonal plasma cells grow abnormally and uncontrollably in the bone marrow and produce monoclonal immunoglobulin, causing hypercalcemia as well as organ dysfunction, including renal insufficiency, anemia, and destructive bone lesions.^1^, ^2^ In 2020, MM made up 10% of hematological malignancies and ranked third behind non‐Hodgkin lymphoma and leukemia.^1^, ^3^, ^4^ The cause of this malignancy remains unclear, but primary cytogenetic abnormalities are known to contribute to initiating the malignant development, and, together with secondary abnormalities acquired along the disease, dysregulate the cell cycle, leading to cell proliferation and clonal growth.^1^, ^3^ Bone disease is responsible for the severe complications of MM, such as fractures that bring debilitating pain and increase the mortality risk.^5^, ^6^ Bone involvement is caused by myeloma cells infiltrating the bone marrow and releasing osteoclast‐stimulating factors and osteoblast‐inhibiting factors, leading to both increased bone resorption and reduced bone formation, resulting in excessive bone destruction and lytic lesions.^5^, ^6^, ^7^, ^8^ To date, no treatment for myeloma is truly curative, but aims at bringing longer time to relapse and to increase overall survival and quality of life.^1^, ^2^ Cannabinoids are molecules isolated from *Cannabis* plant and are known to act mainly through modulation of cannabinoid receptors, such as cannabinoid receptor 1 (CB1) and 2 (CB2), and cannabinoid‐like receptors, such as some members of the transient receptor potential (TRP) channels.^9^ The main biological properties studied for phytocannabinoids, are their anti‐inflammatory and analgesic effects, supported by preclinical and preliminary clinical data.^9^ Cannabinoids are already used in cancer patients who receive chemotherapy and radiotherapy for their palliative properties, such as analgesic, antinauseant, antidepressant, and antiemetic effects.^9^ However, in recent years, more and more studies have analyzed the proper anticancer effect of cannabinoids.^9^, ^10^ In fact, cannabinoids were evidenced to influence tumor cell growth, by inhibition of proliferation, block of cell cycle, induction of autophagy and apoptosis, inhibition of cancer cell invasion and metastasis, inhibition of angiogenesis and interaction with the immune system.^9^, ^10^, ^11^, ^12^, ^13^, ^14^ The anticancer effect of cannabinoids has been demonstrated in many cancer models such as breast, lung, prostate, testicular, gastric, pancreatic, skin, colon, bone cancer, d glioblastoma, lymphoma, leukemia, neuroblastoma, *in vitro* and in some *in vivo* models.^9^, ^13^ Additionally, clinical trials are demonstrating that cannabinoids have anticancer properties, and are studying the safety of these compounds.^15^, ^16^, ^17^, ^18^ For example, a clinical trial involving patients with recurrent glioblastoma, showed a 1‐year survival in 83% of patients treated with nabiximols (standardized extract of *Cannabis sativa* L.) plus temozolomide compared to 44% of patients treated with temozolomide alone, and an overall survival at 2 years in 50% of patients in the first group versus 22% in the second one.^17^ Research on cannabinoids and MM is limited.^19^ There have been a few studies showing that some of them inhibit tumor cell growth by blocking the cell cycle and causing cell death in MM cell lines, while they did not cause cytotoxicity in non‐tumor cells. Moreover, they have been found to synergize with chemotherapeutic drugs or proteasome inhibitors overcoming drug‐resistance. The only *in vivo* study showed that a synthetic cannabinoid agonist suppressed tumor growth in a murine model of MM.^20^, ^21^, ^22^, ^23^, ^24^, ^25^ Scientific literature also reported the beneficial effect of cannabinoids against bone disease.^26^ Indeed, CB2 agonists prevented pathological bone fractures caused by cancer‐induced osteolytic destruction.^27^ Moreover, cannabidiol (CBD) improved fracture healing, was involved in collagen cross‐linking and stabilization^28^ and reduced bone resorption *in vivo* in mice.^29^ While Δ^9^‐tetrahydrocannabinol (THC) and CBD have been the phytocannabinoids most studied for their anticancer effects, less is known about the minor phytocannabinoids, such as cannabigerol (CBG), cannabichromene (CBC), cannabinol (CBN), and cannabidivarin (CBDV). The term minor phytocannabinoids is linked to their lower abundance relative to THC and CBD in medical cannabis.^30^ Only preliminary evidences have been published for them, regarding promising antitumoral effects in human cancers.^30^ So, our study aimed to investigate the potential anticancer properties of CBG, CBC, CBN, and CBDV *in vitro* in three human MM cell lines as well as the anticancer properties of CBN *in vivo* in a murine MM model.

## 2 MATERIALS AND METHODS

### 2.1 Cell lines

U266B1 (U266, RRID:CVCL_0566), RPMI‐8226 (RPMI, RRID:CVCL_0014), and SKO‐007 (SKO, RRID:CVCL_4974) MM cell lines were purchased from ATCC (LGC Standards, Milan, Italy) and cultured in RPMI1640 medium (Lonza, Milan, Italy) supplemented with 10% fetal bovine serum (FBS), 2 mM L‐glutamine, 100 IU/mL penicillin, 100 μg/mL streptomycin, and 1 mM sodium pyruvate. Human osteoblast cell line CI‐huOB (HuOB) was purchased from InSCREENex GmbH (Braunschweig, Germany) and cultured in DMEM glucose high medium (EuroClone, Milan, Italy) supplemented with 100 IU/mL penicillin, 100 mg streptomycin, 10% FBS, 1 mM sodium pyruvate, and 2 mM L‐glutamine. Monocyte THP‐1 (RRID:CVCL_0006) cell line was purchased from Istituto Fondazione di Oncologia Molecolare (IFOM, Rome, Italy), and cultured in RPMI1640 medium supplemented with 10% FBS, 2 mM L‐glutamine, 100 IU/mL penicillin, 100 μg/mL streptomycin, and 0.05 mM β‐mercaptoethanol. THP‐1 cells have been differentiated into osteoclasts. THP‐1 cells were seeded at a density of 2.5 × 10^4^ cells/well in a 96 well plate with phorbol‐12 myristate‐13 acetate (PMA, Sigma‐Aldrich, Milan, Italy) 100 ng/mL to differentiate in macrophages. After 3 days, receptor activator of nuclear factor κ B ligand (RANK‐L, AdipoGen Life Sciences, San Diego, USA) 66 ng/mL and macrophage colony stimulating factor (M‐CSF, BioVision Incorporated, Milpitas, USA) 33 ng/mL were added and changed every 3–4 days to differentiate cells in osteoclasts. After 14 days, tartrate‐resistant acid phosphatase (TRAP) staining was performed using Leukocyte Acid Phosphatase kit (Sigma‐Aldrich, Milan, Italy) to identify osteoclasts (cells containing wine‐red particles (TRAP‐positive) and multinucleated). A 37°C temperature, 5% CO_2_ content, and 95% humidity were maintained for all cell lines. Cells were authenticated by Short Tandem Repeat (STR) DNA Genotype analysis and Cellosaurus database (https://www.cellosaurus.org)^31^ comparison within the last 3 years. The experiments were conducted with mycoplasma‐free cells.

### 2.2 Reagents

Pure CBG, CBC, CBN, and CBDV were purchased by Cayman Chemicals (Ann Arbor, Michigan, USA). Compounds were dissolved in ethanol 70% at 50 mM, aliquots were stored at −20°C and each aliquot was used one time.

### 2.3 Cell viability assay

Cells were seeded at a density of 3 × 10^4^ cells/mL in 96‐well plates, in a final volume of 100 μL/well. After 72 h of treatment, cell viability was assessed by adding 3‐[4,5‐dimethylthiazol‐2‐yl]‐2,5 diphenyl tetrazolium bromide (MTT, 0.8 mg/mL) (Sigma Aldrich, Milan, Italy) to the media. Six replicates were used for each treatment. After 3 h, salt crystals were solubilized with dimethyl sulfoxide (DMSO). The absorbance of the samples against a background control was measured by ELISA reader microliter plate μQuant (BioTek Instruments, Winooski, VT, USA). All experiments were repeated three times.

### 2.4 Cell death assay

To evaluate cell death, Annexin V‐FITC and propidium iodide (PI) staining was used. Cells were seeded at a density of 3 × 10^4^ cells/mL in 6‐well plates and after 1 day of incubation treatments were added. 48 h post‐treatment, cells were stained with 5 μL of Annexin V‐FITC (Vinci Biochem, Vinci, Italy) for 10 min at room temperature, washed once with binding buffer (10 mM N‐[2‐hydroxyethyl] piperazine‐N′‐[2‐ethanesulfonic acid] (HEPES)/NaOH, pH 7.4, 140 mM NaCl, 2.5 mM CaCl_2_), then stained with 20 μg/mL PI (Sigma‐Aldrich, Milan, Italy) and analyzed on a FACScan flow cytometer using CellQuest software (BD Biosciences, San Jose, CA, USA). All experiments were repeated three times.

### 2.5 Western blot analysis

Cell lysates obtained with lysis buffer (composed by TRIS 1 M pH 7.4, NaCl 1 M, EGTA 10 mM, NaF 100 mM, deoxycholate 2%, EDTA 100 mM, TritonX‐100 10%, glycerol, SDS 10%, Na_2_P_2_O_7_ 1 M, Na_3_VO_4_ 100 mM, PMSF 100 mM, cocktail of enzyme inhibitors) were separated on a SDS polyacrylamide gel, transferred onto Hybond‐C extra membranes (GE Healthcare, Chicago, IL, USA) and blocked with 5% low‐fat dry milk in phosphate‐buffered saline 0.1% Tween 20. Each membrane was immunoblotted with specific antibodies: mouse anti‐glyceraldehydes‐3‐phosphate dehydrogenase (GAPDH, 1:1000, sc‐47724 Santa Cruz Biotechnology, Heidelberg, Germany), rabbit anti‐phospho‐histone H2AX (Ser139) (1:1000, #9718 Cell Signaling Technology, Danvers, MA, USA) and then incubated with their respective HRP‐conjugated anti‐rabbit or anti‐mouse (1:2000, #7074, #7076 Cell Signaling Technology, Danvers, MA, USA) Abs. Peroxidase activity was visualized with the LiteAblot®PLUS or TURBO (EuroClone, Milan, Italy) kit and densitometric analysis was carried out by ChemiDoc XRS+ using the Quantity One software version 4.6 (Bio‐Rad, Milan, Italy). All experiments were repeated three times.

### 2.6 Cell invasion assay

The invasion assay was performed using the Corning® BioCoat™ Matrigel® Invasion Chamber (Corning, NY, USA), whose inserts are pre‐coated with extracellular matrix (ECM) proteins. 1 × 10^4^ HuOB cells were seeded in the bottom chamber. The day after, MM cells at a density of 2.5 × 10^4^ cells/well were pre‐treated with 1 μM calcein‐AM (Life Technologies, Monza, Italy) for 30 min and then transferred inside the invasion chamber. Treatments were added in the upper part for 24 h. One well without HuOB was used as negative control. Images of migrated cells in three randomly selected fields were captured and evaluated under fluorescent microscopy (LeitzFluovert FU, Leica Microsystems, Wetzlar, Germany).

### 2.7 Bone resorption assay

THP‐1 cells were seeded in a 96 well plate on bovine bone slices (Boneslices.com, Jelling, Denmark) and differentiated in osteoclasts as previous described. After 7 days, treatments in new media were added every 3–4 days (day 7, 10). The supernatants were collected (day 10 and 14) and the release of the C‐terminal type I collagen fragments was evaluated by ELISA (Human Cross‐linked C‐telopeptides of Type I Collagen, CICP ELISA Kit, Novatein Biosciences, Woburn, MA, USA) to quantify the bone resorption. The absorbance of the samples against a background control was measured by ELISA reader microliter plate μQuant (BioTek Instruments, Winooski, VT, USA). All experiments were repeated three times.

### 2.8 Treatment on a xenograft model of MM

The effect of CBN was tested *in vivo* in a xenograft model of MM, derived from the inoculation of U266 cells. All the procedures involving the animals were conducted by MTTlab Srl (Trieste, Italy) according to the guidelines of Ministry of Health (DDL 116 of February 21, 1992 and subsequent amendments), to the Guide for the Care and Use of Laboratory Animals, Department of Health and Human Services publication no. 86‐23 (National Institutes of Health, Bethesda, MD, 1985) and to the approved experimental protocol procedure (Authorization no. 625/2021‐PR released in accordance with article 31 D.lsg 26/2014). Ten female B‐NDG mice (NOD‐Prkdcscid IL2rgtm1/Bcgen) aged 11 weeks were provided by Envigo Italy. Animal were maintained under a daily 12 h photoperiod in controlled cabinet. Following a period of acclimatation, 5 × 10^6^ U266 cells were subcutaneous inoculated on the hip of the mice. Treatments started when tumors were palpable, 10 days after inoculation (day 0). Animals were divided into two groups (*n* = 5 per group): (1) control (CTRL) received ethanol 70% 50 μL; (2) CBN received cannabinol 15 mg/kg. Treatments were administered subcutaneously every 3 days for 3 weeks, for a total of 7 treatments (day 0, 3, 6, 9, 12, 15, and 18). The body weight of the animals was measured before the starting of the treatment and every 3 days. At the end‐point, mice were sacrificed and macroscopic necroscopy was performed. Moreover, all the tumors were explanted, weighted, along with liver, spleen, and pancreas.

### 2.9 Statistical analysis

Statistical analysis was achieved with GraphPad Prism 9.0.1(128) software (GraphPad Software, San Diego, CA, USA). The data presented represent the mean with standard deviation (SD) of three independent experiments. *p*‐Values <0.05 are considered statistically significant. One‐way ANOVA followed by Dunnett's multiple comparison post‐test was used for in vitro analysis, while Mann–Whitney was used for in vivo studies.

## 3 RESULTS

### 3.1 CBG, CBC, CBN, and CBDV induced cell growth inhibition in human MM cell lines

**FIGURE 1:**
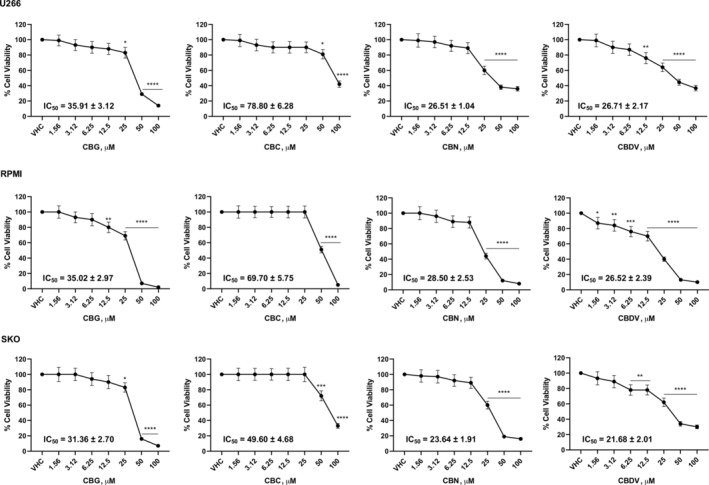
CBG, CBC, CBN, and CBDV effect on MM cell viability. U266, RPMI, and SKO cells were treated for 72 h with different doses of CBG, CBC, CBN, and CBDV. Cell viability was determined by MTT assay. Data shown are expressed as the mean ± SD of three separate experiments. *p < 0.05, **p < 0.01, ***p < 0.001, ****p < 0.0001 versus VHC.

The effect of CBG, CBC, CBN, and CBDV on viability of three MM cell lines was evaluated by MTT assay. Cells were treated with different doses of vehicle (VHC) or phytocannabinoids up to 100 μM. Results show that all of them reduced MM cell viability with different efficacy. IC_50_ value, indicated that CBN and CBDV were the most efficacious in reducing cell viability, followed by CBG and finally by CBC, that was the least effective (Figure 1).

### 3.2 CBG, CBC, CBN, and CBDV induced cell death in human MM cell lines

**FIGURE 2:**
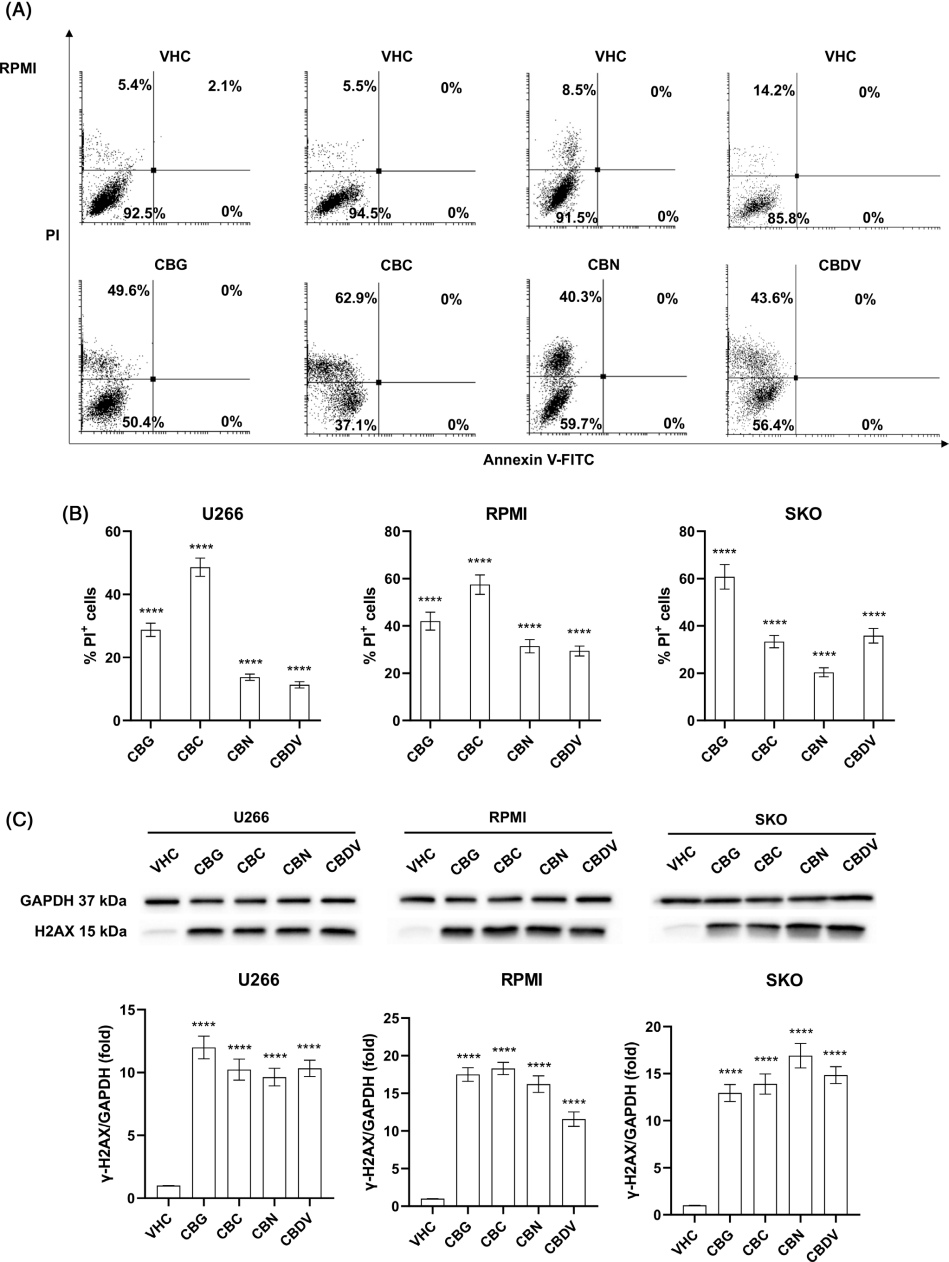
CBG, CBC, CBN, and CBDV effect on cell death in MM cell lines. MM cells were treated for 48 h with CBG, CBC, CBN, or CBDV. (A, B) Cell death was determined by Annexin V‐FITC/PI staining and cytofluorimetric analysis. (A) Histograms are representative of three experiments in RPMI cells. (B) Percentage of PI^+^ cells compared to VHC. Data shown are expressed as mean ± SD of three separate experiments. (C) Effect of cannabinoids in the modulation of γ‐H2AX protein on MM cell lines treated as above described. The expression of γ‐H2AX was determined with western blot analysis. GAPDH was used as loading control to normalize densitometric values for γ‐H2AX. A representative image is shown for one of three experiments. Folds (mean ± SD of three experiments) are changes respect to vehicle. ****p < 0.0001 versus VHC.

To better investigate phytocannabinoids‐induced growth inhibition, Annexin‐V/PI staining followed by flow cytometry was used to evaluate cell death on the three MM cell lines. Cells were treated with vehicle or the IC_50_ dose for each phytocannabinoid and after 48 h the cell death assay was performed. Results show that CBG, CBC, CBN, and CBDV induced necrotic cell death, as seen by the increased % of PI positive cells in treatments compared to vehicle (*p* < 0.0001) (Figures 2A,B; S1). This effect was further supported by western blot analysis. Indeed, the expression of γ‐H2AX protein, marker of DNA damage, was statistically increased (*p* < 0.0001) following treatment with CBG, CBC, CBN, and CBDV, confirming MM cell death (Figure 2C).

### 3.3 Effect of CBG, CBC, CBN, and CBDV in HuOB cells

**FIGURE 3:**
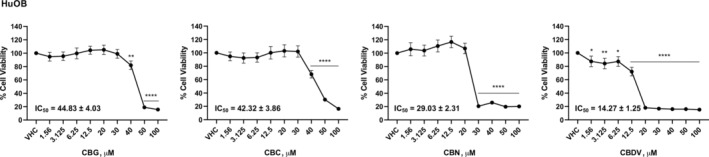
CBG, CBC, CBN, and CBDV effect on HuOB cell line viability. HuOB were treated for 72 h and then MTT assay was used to determine cell viability. Three separate experiments are shown as mean ± SD. *p < 0.05, **p < 0.01, ****p < 0.0001 versus VHC.

Given that bone lesions, caused by cancer cells infiltrating the bone marrow, are one of the main complications in MM patients, the effect of phytocannabinoids in interfering with the bone–MM cells interaction was investigated. So, first, the effect of different doses of CBG, CBC, CBN, and CBDV in reducing cell viability of HuOB was evaluated. Results show that all the phytocannabinoids reduced cell viability with different efficacy (Figure 3). The doses 12.5 μM for CBG, CBC, CBN, and 6.25 μM for CBDV, were selected for bone–MM cell interaction study, due to their noncytotoxic effects in HuOB and MM cell lines.

### 3.4 CBG, CBC, CBN, and CBDV reduce the invasion of MM cells toward HuOB cells

**FIGURE 4:**
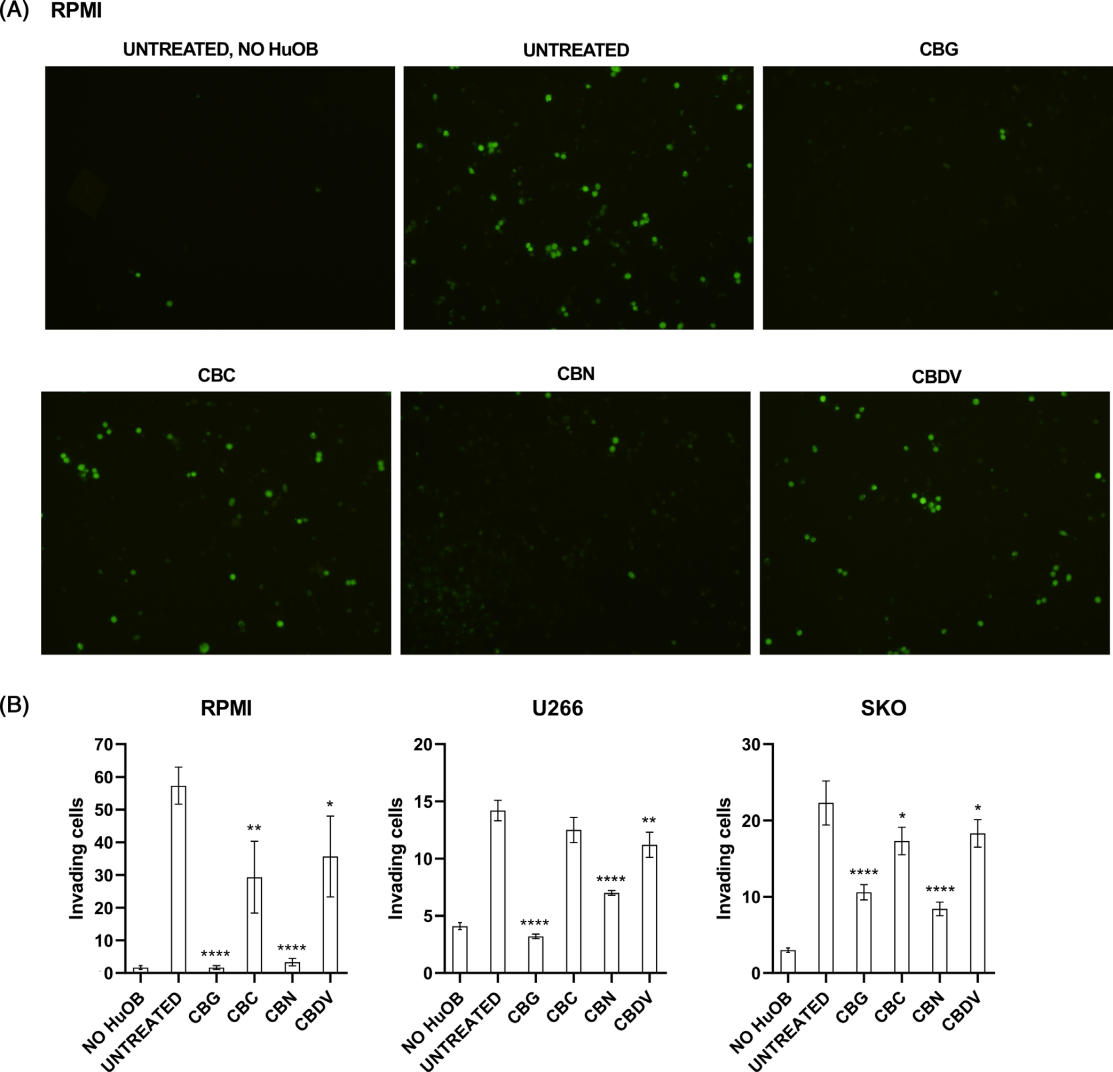
MM cell invading the ECM covered membrane toward HuOB cells, after treatment with CBG, CBC, CBN, and CBDV. (A) Representative image of calcein stained RPMI cells invading the ECM coated membrane. Three random fields were observed under a fluorescence microscope. Magnification 10×. (B) The number of invading cells represent the mean ± SD of three separated experiments. *p < 0.05, **p < 0.01, ****p < 0.0001 versus UNTREATED.

To assess if phytocannabinoids modulate the bone–MM interaction, the invasion of MM cells toward HuOB cells was evaluated. So, an invasion assay was performed using ECM coated transwells. HuOB were plated on the bottom chamber, while MM cells, pre‐treated with calcein, were plated on the upper chamber. Treatments (12.5 μM for CBG, CBC, CBN, and 6.25 μM for CBDV) were added on the upper chamber. Results show that after 24 h, in absence of HuOB the MM cells invasion in lower chamber did not occur, while the presence of HuOB acted as a chemoattractant. In fact, it was already found that osteoblasts promote migration and invasion of myeloma cells.^7^ The treatments with the four phytocannabinoids reduced the number of MM cells that invaded the ECM coated membrane, but in particular CBG and CBN were more effective (*p* < 0.0001) (Figure 4).

### 3.5 CBG, CBC, CBN, and CBDV reduce the bone resorption

**FIGURE 5:**
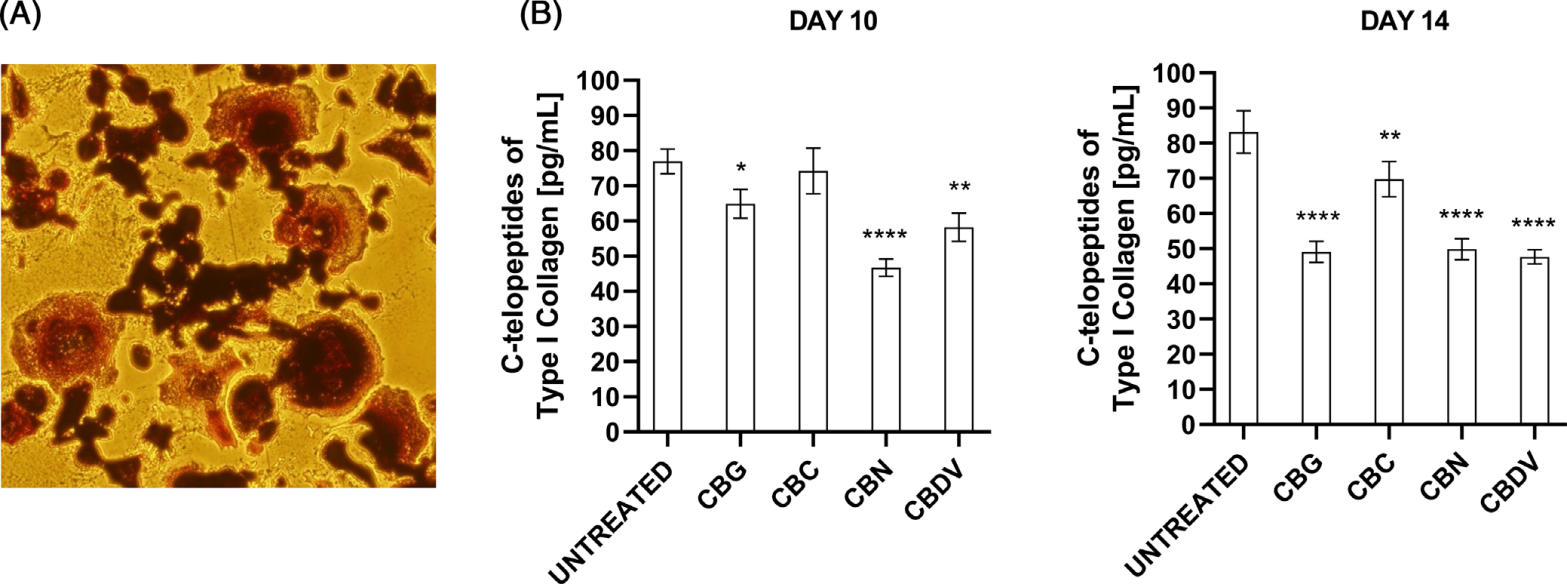
Effect of CBG, CBC, CBN, and CBDV on bone resorption. (A) Representative images of osteoclasts differentiated from THP‐1 cells (TRAP‐positive). Magnification 20×. (B) Effect of CBG, CBC, CBN, and CBDV on modulating the release of C‐terminal type I collagen fragments, from bovine bone slices, by osteoclasts. The release of C‐terminal type I collagen fragments was evaluated by ELISA. Results are the mean ± SD of three experiments. *p < 0.05, **p < 0.01,  ****p < 0.0001 versus UNTREATED.

The following step was to investigate if phytocannabinoids also modulate the bone resorption. In fact, lytic bone lesions in MM patients are due to the over activation of osteoclast cells, induced by MM cells. So, we performed a bone resorption assay, evaluating the release of C‐terminal type I collagen fragments from bovine bone slices by osteoclasts through ELISA assay. In fact, N‐terminal and C‐terminal cross‐linked telopeptides of type I collagen breakdown products of osteolysis are used as biomarkers.^6^ Briefly, THP‐1 cells were seeded in a 96 well plate on bovine bone slices and differentiated in osteoclasts (Figure 5A). After 7 days, treatments (12.5 μM for CBG, CBC, CBN, and 6.25 μM for CBDV) were added every 3–4 days. The supernatants were collected at day 10 and 14 and the release of the C‐terminal type I collagen fragments was evaluated by ELISA. Results in Figure 5B show that CBG, CBN, and CBDV reduced these fragments already after 10 days, meaning a reduction of bone resorption. After 14 days, all of them were effective, mainly CBG, CBN, and CBDV (*p* < 0.0001).

### 3.6 CBN reduced tumor mass in a xenograft model of MM

**FIGURE 6:**
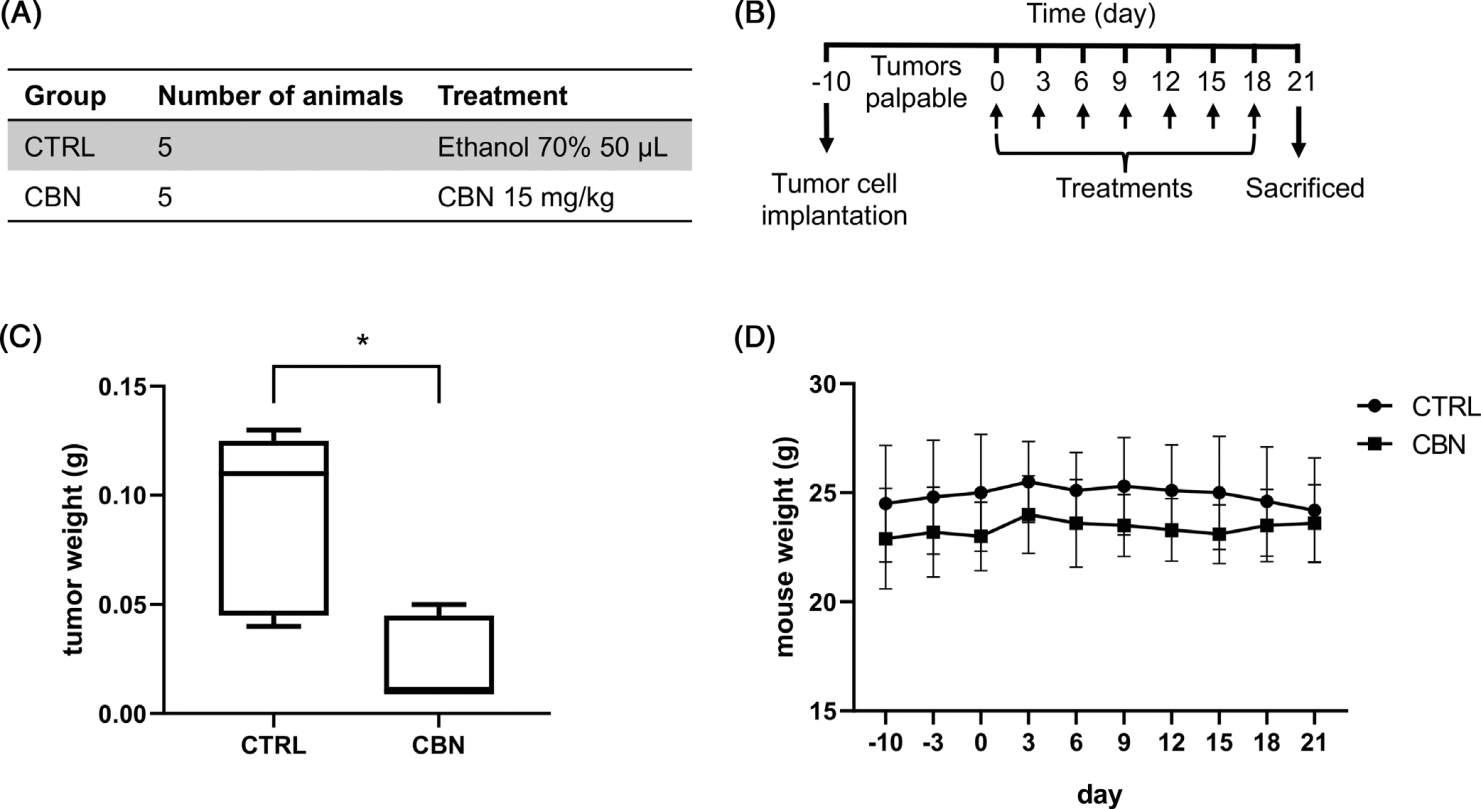
CBN effect on a xenograft model of MM. (A) Groups of animals and treatments received. (B) Experimental design of animal treatments. When tumors became palpable, animals were treated subcutaneous every 3 days, for 3 weeks. (C) Tumor weight at the end of treatments. Box spans from the first to the third quartile. The line inside the box indicates the median. The whiskers extend either to the minimum/maximum data value. *p < 0.05. (D) Body weight of the mice during the treatment. The graph shows the mean ± SD from five animals for group.

For a preliminary study, a xenograft model of MM was used to evaluate the anticancer effect of CBN which proved to be the most effective in the previous experiments regarding inhibition of MM cell growth, cell invasion, and bone resorption. The xenograft model of MM was obtained by subcutaneous inoculation of U266 MM cells on mice. When tumors were palpable, 10 days after inoculation (day 0), animals were treated by subcutaneous injection every 3 days for 3 weeks, with ethanol 70% 50 μL (control group, CTRL) or CBN 15 mg/kg (group CBN) (Figure 6A,B). At the end‐point mice were sacrificed and macroscopic necroscopy was performed. All the tumors were explanted and weighted (Figure 6C), along with liver, spleen, and pancreas. Results showed that, after 3 weeks of treatment, a significant reduction (*p* = 0.0397) of tumor weight was observed in mice treated with CBN, respect to the control group (Figure 6C). For a toxicological evaluation, the body weight of the animals was measured every 3 days during the treatment. Results showed that the body weight of animals in CBN group was like the CTRL group (Figure 6D) and as compared to the CTRL group, CBN animals' initial and final body weights did not differ statistically (Table S1). Furthermore, the CBN group did not differ statistically from the CTRL group in terms of liver, spleen, and pancreas weight (Table S1).

## 4 DISCUSSION

Despite many treatment options available, there is a need for new treatments for MM patients who became refractory to all options.^1^, ^3^ Cannabinoids or medical *Cannabis* extracts are used in cancer patients who receive chemotherapy and radiotherapy for their palliative properties, like analgesic, antinauseant, antiemetic, and antidepressant properties, but they are also demonstrating direct anticancer effect^9^, ^10^ in preclinical cancer models and in clinical trials.^30^ In MM, it was already studied the effect of CBD, THC, the synthetic cannabinoid agonists WIN‐55, PGN‐6, ‐17, ‐34, and ‐72, the inverse agonist of CB_2_ phenylacetylamide (PAM), and β‐caryophyllene, which showed promising anticancer effects *in vitro* and, WIN‐55, *in vivo* in animal models.^19^ The activity of cannabinoids in MM was supported by the expression of some cannabinoid target receptors in MM, even if only few information are available. For example, CB2 receptor was showed to be highly expressed in MM cell lines and in CD138^+^ cells from MM patients, while CB1 and transient receptor potential vanilloid type‐2 (TRPV2) were not expressed at appreciable levels in MM cell lines.^20^, ^21^, ^23^, ^24^ Here, we investigated the anticancer effect of four minor phytocannabinoids, CBG, CBC, CBN, and CBDV *in vitro* in three MM cell lines and of CBN *in vivo* in a xenograft murine model of MM. Regarding the cytotoxicity of the phytocannabinoids investigated in this study, we found that they inhibited MM cell growth, in a dose dependent manner. As we can see from the IC_50_, CBN, and CBDV were the most efficacious in reducing cell viability, followed by CBG and in the end by CBC, that was the less cytotoxic in all the three MM cell lines. The phytocannabinoids CBG, CBC, CBN, and CBDV are less studied compared to THC and CBD, and no data were reported for hematological cancers. Anyway, there are evidence for their effect in reducing solid cancer cell growth. For example, CBG reduced viability of human pancreatic ductal adenocarcinoma, breast cancer, prostate cancer, glioblastoma, and glioma stem‐like cells.^32^, ^33^, ^34^, ^35^ For CBC, a reduction of cell growth was evidenced, for example, in mesothelioma, colorectal, and prostate carcinoma cells.^36^, ^37^, ^38^ Few data were reported also for CBN, which was found to reduce viability of human breast cancer, mesothelioma, and prostate carcinoma cells^33^, ^36^, ^38^ and for CBDV, that blocked cancer cell growth of human mesothelioma, colon carcinoma, and prostate carcinoma cells.^36^, ^37^, ^38^ In line with our results of rank of potency, CBN was more efficacious then CBG in human breast cancer cells^33^ and CBC was less potent than CBG and CBDV in reducing cell viability of human colorectal cancer cells.^37^ Nevertheless, the rank of potency of these phytocannabinoids can be different in cells of different tumor models, but also in cell lines derived from the same tumor, as observed in other works.^36^, ^38^ Herein, we demonstrated that the inhibition of MM cells growth was associated with induction of necrotic cell death by CBG, CBC, CBN, CBDV, while as evidenced for CBG or CBN, the main mechanism of cell death was apoptosis in human pancreatic cancer, breast cancer, mesothelioma, cholangiocarcinoma, and glioblastoma cells.^32^, ^33^, ^35^, ^36^, ^39^ However, regarding MM, CBD triggered necrotic cell death in MM cell lines^20^, ^22^ in line with our results. In MM, bone disease is due to myeloma cells infiltrating the bone marrow and inducing excessive bone destruction.^1^, ^7^ Here, we found that CBG, CBC, CBN, and CBDV reduced the invasion of MM cells toward osteoblasts cells, but in particular CBG and CBN were the most effective. Moreover, CBG, CBC, CBN, and CBDV reduced the bone slices resorption by osteoclast, with CBG, CBDV and CBN being the most effective. In accord, studies showed that cannabinoids can regulate osteoclasts, osteoblasts, and adipocytes *in vitro* and *in vivo* ^26^ and, in particular, CB2 receptor agonists reduced cancer‐induced osteolytic destruction^27^ and CBD attenuated stimulatory effects on osteoclast induced by an activator of GPR55, a cannabinoid receptor, and reduced bone resorption *in vivo* in mice via modulation of GPR55 signaling.^29^ Ultimately, we found that CBN reduced tumor mass in a xenograft murine model of MM. Many studies found that cannabinoids reduced tumor growth *in vivo* and CBG, in particular, decreased tumor growth in a mouse model of melanoma^40^ and in a xenograft mouse model of colon adenocarcinoma.^37^ About MM, one research article found that cannabinoid agonist WIN‐55 significantly suppressed tumor growth *in vivo* in a xenograft MM mouse model.^23^ Together, our results suggest that CBG, CBC, CBN, and CBDV can be promising anticancer agents for MM, due to their cytotoxic effects on MM cell lines and, for CBN, in *in vivo* xenograft mouse model of MM, and due to their beneficial effect on the bone in terms of reduction of MM cells invasion toward the bone and bone resorption (mainly CBG and CBN). Further study is needed to better understand how phytocannabinoids work, as well as to better investigate their effects *in vivo*.

## AUTHOR CONTRIBUTIONS

Conceptualization: **Massimo Nabissi**. Data curation: **Cristina Aguzzi**, **Laura Zeppa**. Formal analysis: **Massimo Nabissi**, **Beatrice Morelli**. Funding acquisition: **Massimo Nabissi**, **Sazzad Hossain**. Investigation: **Cristina Aguzzi**, **Laura Zeppa**, **Oliviero Marinelli**. Methodology: **Cristina Aguzzi**, **Laura Zeppa**, **Maria Beatrice Morelli**, **Massimo Nabissi**. Project administration: **Massimo Nabissi**. Resources: **Massimo Nabissi**. Supervision: **Maria Beatrice Morelli**, **Massimo Nabissi**. Validation: **Maria Beatrice Morelli**, **Massimo Nabissi**. Visualization: **Cristina Aguzzi**, **Laura Zeppa**, **Maria Beatrice Morelli**. Writing‐original draft: **Cristina Aguzzi**, **Laura Zeppa**, **Massimo Nabissi**. Writing‐review & editing: **Maria Beatrice Morelli**, **Consuelo Amantini**, **Martina Giangrossi**, **Giorgio Santoni**. The work reported in the paper has been performed by the authors, unless clearly specified in the text.

## CONFLICT OF INTEREST STATEMENT

Sazzad Hossain is CEO of Entourage Biosciences Inc., Vancouver, V6E 4N7, Canada. The other authors declare no conflicts of interest.

## Supporting information


**FIGURE S1.** CBG, CBC, CBN, and CBDV effect on cell death in MM cell lines. MM cells were treated for 48 h with CBG, CBC, CBN, or CBDV. Cell death was determined by Annexin V‐FITC/PI staining and cytofluorimetric analysis. Histograms are representative of three experiments in U266 and SKO cells.

**TABLE S1.** Initial and final body weight of mice; liver, spleen, and pancreas weight at the end of treatment. Results are expressed as the mean ± SD from five animals for group.

## Data Availability

The data that support the findings of this study are presented in the main text article and in the online supplementary information file.
