# Supplementary figures and images for: Anticancer effect of minor phytocannabinoids in preclinical models of multiple myeloma
Source: Biofactors. 2024 May 17;50(6):1208–19. doi: 10.1002/biof.2078 (PMC11627469; doi:10.1002/biof.2078)

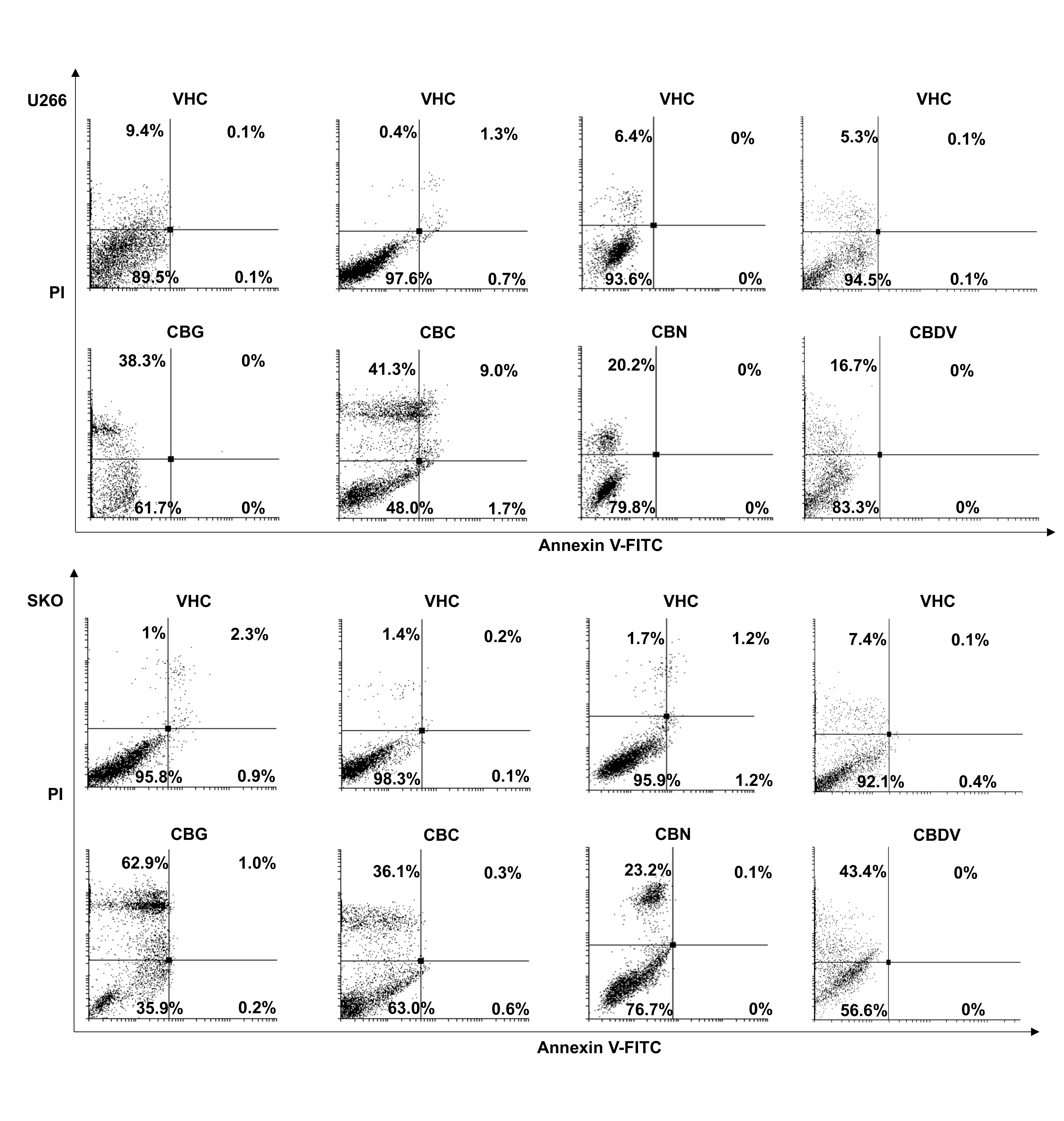

Supplement: Supplementary file 1 — FIGURE S1. CBG, CBC, CBN, and CBDV effect on cell death in MM cell lines. MM cells were treated for 48 h with CBG, CBC, CBN, or CBDV. Cell death was determined by Annexin V‐FITC/PI staining and cytofluorimetric analysis. Histograms are representative of three experiments in U266 and SKO cells. [file BIOF-50-1208-s001.tif]
