# Supplementary material for: Anticancer effect of minor phytocannabinoids in preclinical models of multiple myeloma
Source: Biofactors. 2024 May 17;50(6):1208–19. doi: 10.1002/biof.2078 (PMC11627469; doi:10.1002/biof.2078)
Supplement: Supplementary file 2 — TABLE S1. Initial and final body weight of mice; liver, spleen, and pancreas weight at the end of treatment. Results are expressed as the mean ± SD from five animals for group. [file BIOF-50-1208-s002.docx]

| **Group** | **Initial body weight (g)** | **Final body weight (g)** | **Liver weight (g)** | **Spleen weight (g)** | **Pancreas weight (g)** |
| --- | --- | --- | --- | --- | --- |
| CTRL | 25 ± 2.68 | 24.2 ± 2.39 | 1.03 ± 0.02 | 0.06 ± 0.02 | 0.14 ± 0.02 |
| CBN | 23 ± 1.60 | 23.6 ± 1.80 | 1.07 ± 0.10 | 0.10 ± 0.03 | 0.13 ± 0.07 |
